# Xiphoid Process Variations: A Review with an Extremely Unusual Case Report

**DOI:** 10.7759/cureus.1613

**Published:** 2017-08-27

**Authors:** Faizullah Mashriqi, Anthony V D'Antoni, R. Shane Tubbs

**Affiliations:** 1 Department of Molecular, Cellular, and Biomedical Sciences, CUNY School of Medicine; 2 Department of Molecular, Cellular and Biomedical Sciences, CUNY School of Medicine; 3 Neurosurgery, Seattle Science Foundation

**Keywords:** xiphoid process, sternum, epigastric mass, bifid, variation

## Abstract

The xiphoid process is a small bony feature of the anterior thoracic wall just inferior to the sternum corpus. Although the xiphoid process is commonly represented as a straight, fully ossified bone in educational textbooks, reports of anomalous processes flood the literature. The xiphoid process can be broad, thin, monofid, bifid, trifid, curved, or deflected and contain foramina. Variations can be mistaken for epigastric masses. Herein, we report an extremely unusual bifid xiphoid process that is deflected anteriorly. This case is discussed in the context of the misdiagnosis of xiphoid process variations and its importance to the clinician.

## Introduction

The xiphoid process (also known as the xiphisternum) is located in the epigastrium region of the anterior thoracic wall. The xiphoid process articulates with the superiorly located sternum corpus at the xiphisternal joint. During the first half of life, this joint is categorized as a symphysis but eventually becomes a synostosis at ~40 years [1]. Anteriorly, the xiphoid process serves as the attachment point for fibers of the rectus abdominis muscle and the aponeurosis of the internal and external oblique muscles of the anterior abdominal wall [2]. The xiphoid process attaches to the linea alba inferiorly and the diaphragmatic slips, transversus thoracis, and costoxiphoid ligaments posteriorly [1-2]. At birth, the xiphoid process is a cartilaginous structure and ossification begins at around three years of life from the superior-most portion [2]. The perforating branches of the internal thoracic artery perfuse the entire sternum.

Understanding the variants of regional bones during invasive procedures or during the interpretation of radiological images is necessary for good clinical care. Anteriorly deflected xiphoid processes can be mistaken for epigastric masses. Moreover, there have been reports of cardiac tamponade in patients with xiphoid foramina following acupuncture procedures that pierced the pericardial sac [1]. We present here a very unusual cadaveric case with an anteriorly deflected xiphoid process and review the literature regarding other malformations of this bony protuberance.

## Case presentation

**Figure 1:**
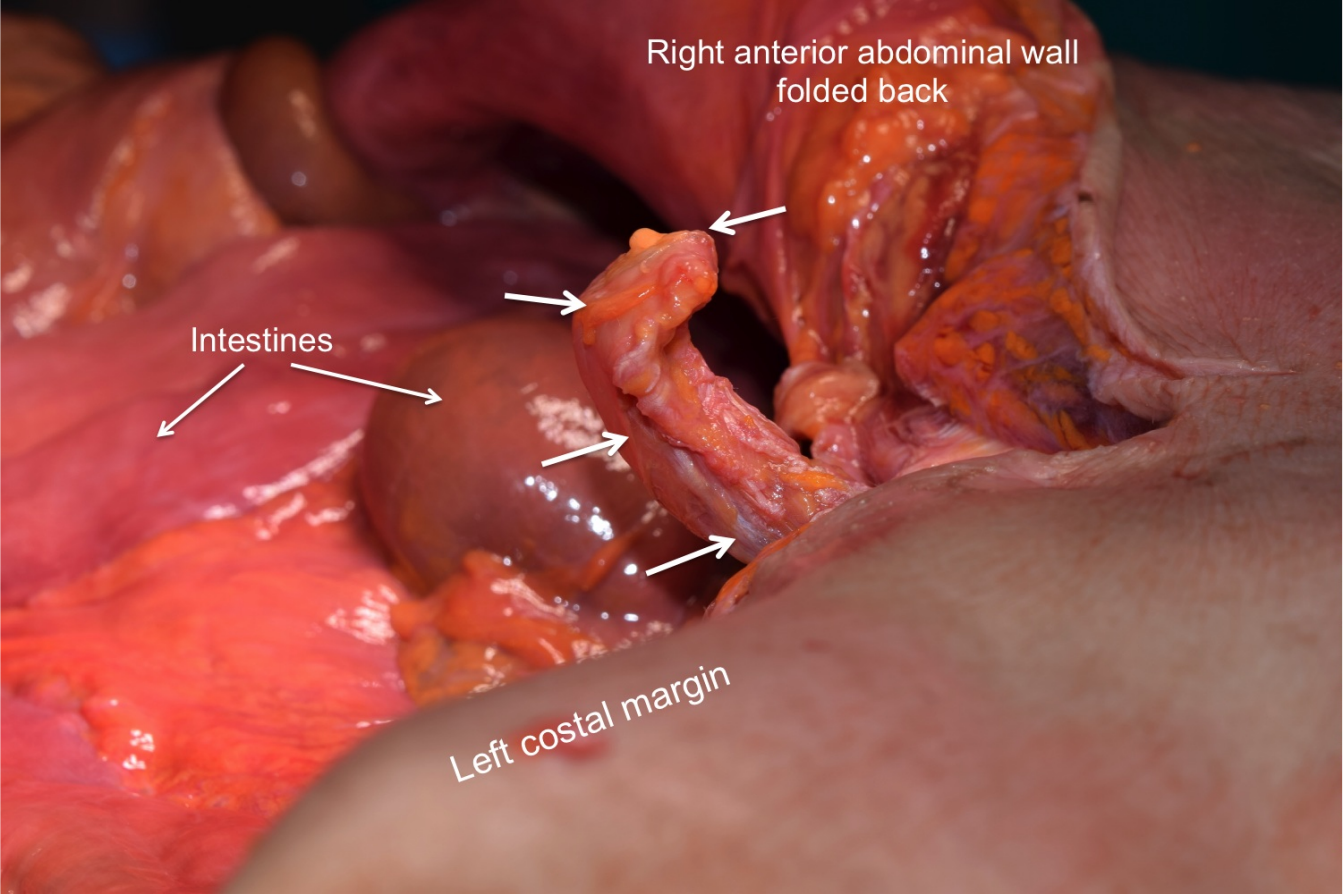
Superolateral view of the xiphoid process identified during routine anatomical dissection No signs of previous trauma or surgery were identified around the xiphoid process. Note the acute angulation of the xiphoid process with a protuberance anteriorly. Arrows outline the congenitally deformed xiphoid process.

During a routine dissection of the abdominothoracic region in an adult male cadaver, aged 78 years at death, an unusual protuberance in the epigastrium was observed. The cause of death of the specimen was kidney failure with a history of diabetes and peripheral vascular disease. No signs of trauma, either recent or old, were identified in the area of the protuberance. Additionally, no signs of past surgical intervention to the region were observed. With continued deeper dissection, a 3 cm bony prominence was exposed. This was found to be continuous and an anterior extension of the xiphoid process (Figure 1). The linea alba was seen to be tented more superficially and was attached at the tip of the abnormally shaped xiphoid process. No other pathology of the abdominal region was noted other than a small left inguinal hernia, which was easily reduced.

## Discussion

The etymology derives from the Greek word “xiphos” meaning straight sword [1], but this is a misnomer, as the xiphoid process is the most variable sternal element and regularly takes on many forms. The xiphoid process can be broad, thin, monofid, bifid, trifid, curved, deflected, and contain foramina [1-3]. Current textbooks and human anatomy atlases depict the xiphoid process as a single, straight, fully ossified bone on the inferior aspect of the sternum corpus. In actuality, significant variation is reported in the literature [1-4].

The variation in xiphoid morphology was demonstrated using multidetector computed tomography (MDCT) in a 500 patients sample [2]. This study found that only 33.2% of patients had a xiphoid process that was in the same plane as the sternum corpus. Ventrally deflected processes were present in 65.4% of the patients. Monofid, bifid, and trifid processes were found in 62.6%, 32.8%, and 4.6% of the patients, respectively. Approximately three percent of xiphoid processes were found to curve at the end, either dorsally or ventrally, and resembled a hook. A unique finding of this study was a reverse “S” shaped xiphoid process, which was found in <1% of patients [2]. The prevalence of these different xiphoid process types is more or less consistent in the literature [3].

Reports of xiphoid foramina flood the literature [1-4]. Xiphoid foramina were present in 43.2% of the patients in one study [2] and in 27.4% of patients in a second study [3]. Most subjects had one xiphoidal foramen, less than seven percent of the subjects had two xiphoidal foramina, and less than two percent of the subjects had three xiphoidal foramina [2]. An interesting case report from India showed a large (1.6 cm by 1.4 cm) foramen on a non-ossified xiphoid process that was pear shaped [1]. Pseudoforamina are another common reporting in the literature for the xiphoid process. A pseudoforamen represents the incomplete fusion of the xiphoid process to the sternum corpus. One study reported 3.6% of patients with a pseudoforamen [3] and a second study reported 1.2% of patients with the same anomaly [2]. Finally, the calcification of the costoxiphoid ligaments occurs in ~10% of patients and was mostly identified in patients over 50 years of age [3].

## Conclusions

Clearly, anomalies in the xiphoid process are common, and significant interindividual variation should be expected. The close proximity of the xiphoid process to thoracic structures and abdominal structures requires a thorough understanding of these easily misdiagnosed variations. We reported here a highly unusual bifid xiphoid process with an anterior deflection, which presented as an unusual epigastric protuberance prior to dissection.
